# Why do women not prepare for pregnancy? Exploring women’s and health care providers’ views on barriers to uptake of preconception care in Mana District, Southwest Ethiopia: a qualitative study

**DOI:** 10.1186/s12884-020-03208-z

**Published:** 2020-09-01

**Authors:** Firanbon Teshome, Yohannes Kebede, Fira Abamecha, Zewdie Birhanu

**Affiliations:** Department of Health, Behavior and Society, Faculty of Public Health, Jimma University, Jimma, Ethiopia

**Keywords:** Preconception care, Barriers, Uptake, Jimma, Ethiopia

## Abstract

**Background:**

Preconception care has the potential to reduce maternal and child morbidities and mortalities. It is a window of opportunity to timely alter or eliminate risk factors for adverse pregnancy outcomes. However, despite strong evidence on the effectiveness of preconception care in safeguarding maternal and child health, its uptake remains low. Therefore, this study aimed to explore barriers to the uptake of preconception care.

**Methods:**

A descriptive qualitative study was conducted in Mana district, Jimma Zone, Oromia region, Southwest Ethiopia from March 02 to April 10, 2019. A purposive sampling approach was used, and 13 key informant interviews (6 in rural and 7 in urban areas) were held with women of different age groups, health extension workers, and health care providers of different professions. In addition, 4 focused group discussions with women of reproductive age groups (two with rural women only and two with urban women only) were conducted. The data were collected by trained experts using semi-structured guides. An inductive process of thematic analysis was employed and the data were coded, categorized, and thematized using Atlas ti version 7.0.71 software.

**Results:**

Four women of reproductive age groups, 1 older woman (grandmother), 2 health extension workers, and 6 health care providers of different professions were interviewed. In addition, a total of 38 women of reproductive age groups participated in the 4 focused group discussions: 20 in the two rural-focused group discussions and 18 in the two urban-focused group discussions. The findings indicated the presence of many barriers affecting the uptake of preconception care and organized into five themes: women-related barriers, husband-related barriers, community-related barriers, health-service-related barriers, and media-related barriers.

**Conclusions:**

This study found a diverse array of potentially modifiable barriers to the uptake of preconception care. The findings imply the importance of scaling up health education and counseling, establishing preconception care strategies and functional units that can address all the components at all levels of health care facilities. Therefore, we recommend all stakeholders, such as program planners and managers, non-governmental organizations, media personnel, and health care providers to work in collaboration to increase the uptake of preconception care.

## Background

Preconception care (PC) is a set of interventions that aim to identify and modify biomedical, behavioral, and social risks to women’s health or pregnancy outcomes through prevention and management [1]. It enables women to realize the importance of optimizing their health before conception [2]. PC is a window of opportunity that aimed to improve women’s health before, during, and after pregnancy through risk assessment, health promotion/counseling, intervention for the identified risks, and if indicated, referral to specialized preconception care (Table 1) [3–5]. Early risk identification and management is the foundation for the health of the mother, her child, and future generations [6].

**Table 1:** Components of preconception care

Components	Examples
**Screening/ Risk assessment**	***Reproductive life plan***: e. g. assess if a woman plans to have children, how long she plans to wait until she becomes pregnant, help her develop a plan
	***Reproductive history***: Check previous adverse pregnancy outcomes e.g. infant death, fetal loss, birth defects, low birth weight, preterm birth
	***Medical history***: Screen for chronic conditions like hypertension, diabetes
	***Medication use***: Review the woman’s current medication use; avoid medications that had an impact on pregnancy unless their benefits out weight risks
	***Infections***: Screen for periodontal, urogenital, and STIs as indicated
	***Genetic screening and family history***: Assess the woman’s risk of chromosomal or genetic disorders.
	***Nutritional assessment***: e.g. assess body mass index, screen for anemia
	***Substance abuse***: Ask the woman about tobacco, alcohol, and drug use
	***Psychosocial concerns***: Screen for depression, anxiety, domestic violence
	***Physical examination***: e.g. pelvic examinations
	***Laboratory testing***: e.g. blood type, screening for HIV/AIDS, hepatitis B
**Counseling/Health promotion**	***Family planning***: Promote family planning for women who are not planning to become pregnant, discuss emergency contraception.
	***Healthy weight and nutrition***: Promote a healthy prepregnancy weight through exercise and nutrition; discuss macro­ micronutrients such as folic acid, iron
	***Healthy behaviors***: Promote healthy behaviors such as nutrition, exercise, safe sex, and discourage risky behaviors such as smoking, alcohol, khat
	***Stress resilience***: e.g. Promote sufficient sleep, relaxation techniques
	***Healthy environments***: Discuss exposures to heavy metals, organic solvents, pesticides, and allergens; give practical tips such as how to avoid exposures.
**Interventions/ Management**	Address the identified medical and psychosocial risks: e.g. folic acid supplementation, and vaccination if indicated, control of pregestational diabetes
	Referral to specialized preconception care if indicated

Worldwide, maternal mortality and morbidity are still major health problems [7], especially in low- and middle-income countries where 99% of all maternal and newborn deaths occur [8]. This can be due to a clear gap in the continuum of care, particularly for women who are not pregnant. They get little attention and enter pregnancy with medical problems and unhealthy behaviors. Ethiopia is also highly suffering from maternal and child mortalities, where the maternal mortality rate was 412 deaths per 100,000 live births [9], under-five mortality rate 55 deaths per 1000 live births, infant mortality rate 43 deaths per 1000 live births and neonatal mortality rate 30 deaths per 1000 live births [10], which is far from achieving the 2020 goal [11]. These problems can be reduced or even prevented through the provision of preconception care.

Despite most of the major fetal organs like the brain and heart are formed during the first few weeks of conception [12], women are often too late to attend antenatal care [13]. For instance, in Ethiopia, the median month for the first antenatal care visit was 4.7 months [9]. This can give rise to several maternal and child adverse pregnancy outcomes. Extending the existing maternal, newborn, and child health continuum of care one step before prenatal care helps to save a number of lives [4].

PC is the only evidence-based intervention aimed at minimizing risks before the crucial time of fetal development [1, 14], which helps to prevent persistent adverse pregnancy outcomes and ensures that mothers and newborns are in the best health possible [14, 15]. However, in many countries, the uptake of preconception care was very low. For example, in France (15.8%) [16], data from the Maryland Pregnancy Risk Assessment Monitoring System (13.5%) [17], Brazil (15.9%) [18], Iran (31.7%) [19], Southern Sri Lanka (27.2%) [20] and Nigeria (2.5%) [21]. In Ethiopia, despite the existence of different opportunities (health extension programs, expansion of health facilities along with human resources, linkage among health facilities, and the existence of community-based health insurance), little is known about PC, and the existing data showed that utilization of PC was very low [22–24]. Therefore, this study aimed to explore barriers to the uptake of preconception care from the perspectives of consumers and providers in the selected district, which helps to inform future interventions for increasing utilization of preconception care.

## Methods

### Study design, setting, and period

A descriptive qualitative study was conducted from March 02 to April 10, 2019, to explore barriers to the uptake of preconception care in the Mana district. The district was purposely selected considering factors like researchers’ familiarity with the study community and resource issues. Mana district is one of the twenty-one districts found in the Jimma zone, Oromia Regional State. It is located 346 km southwest of Addis Ababa (the capital city of Ethiopia), and 22 km from Jimma town. According to the 2019 report obtained from the Mana District Health Office, the district has a total population of 197,911, of which women of reproductive age groups were 43,738 and pregnant women were 6868. The district has a total of 26 Gandas^1^:1 urban Ganda and 25 rural Gandas. It has 7 health centers, 26 health posts, 11 private clinics, and 3 private pharmacies. It also has 68 health extension workers and 121 health care providers of different professions.

### Sampling approaches and study participants

**Fig. 1:**
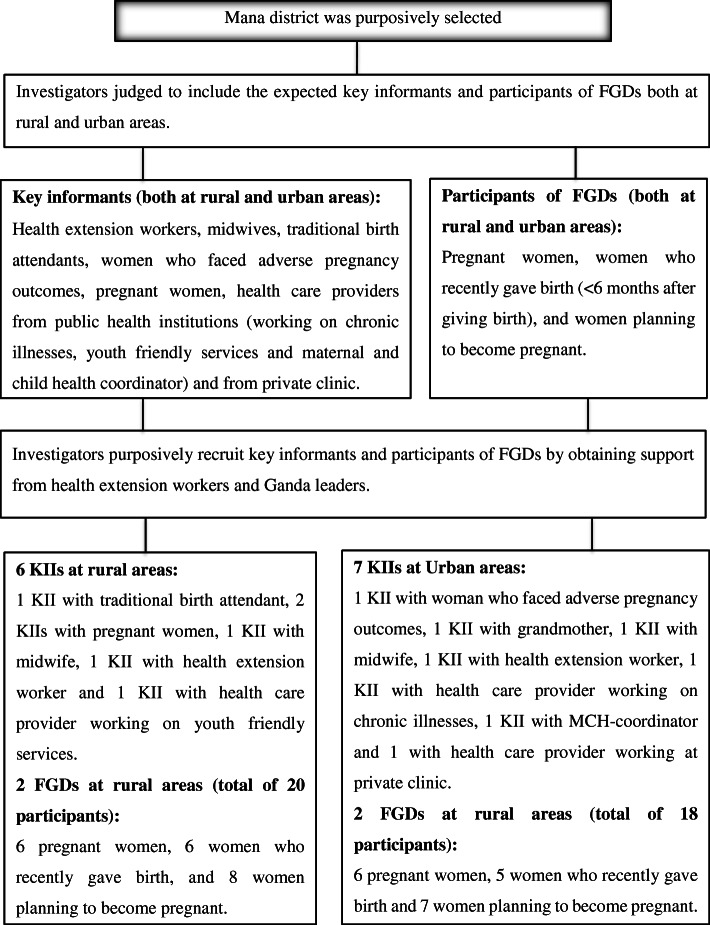
Sampling frame for the study

A purposive sampling technique was employed to recruit key informants and participants of focused group discussions (FGDs) (Fig. 1). The author contacted participants to recruit them, introduce himself, and clarify the reasons for the study, prior to data collection.

Interviews were carried out with community members and health professionals who were expected to be knowledgeable, have experience, or were directly involved in service delivery at the primary health care unit. A total of 13 key informant interviews (KIIs), (6 in rural and 7 in urban areas) were conducted: 1 KII with grandmother, 2 KIIs with pregnant women, 1 KII with women who faced adverse pregnancy outcomes, 1 KII with a traditional birth attendant, 2 KIIs with health extension workers and 6 with health care providers of different professions. The key informants were recruited from both urban and rural areas based on their experiences of giving birth (e.g., grandmother), the chance of getting information about PC (e.g., pregnant women, women who faced adverse pregnancy outcomes), important others (e.g., traditional birth attendant), and health care providers with relevant experiences (health extension workers, maternal and child health co-coordinator, midwives, health care providers working on chronic illnesses, youth-friendly services, and private clinic). Recruitment was held a day before data collection. The site and time of the meeting were decided by considering key informants’ interests. Accordingly, the interviews were conducted at their living home (in areas free from disturbance) with grandmother, pregnant women, women who faced adverse pregnancy outcomes and traditional birth attendant. Similarly, it was conducted at health posts with health extension workers, and at the workplace (in private rooms) with maternal and child health co-coordinator, midwives, health care providers working on chronic illnesses, youth-friendly services, and private clinic.

In addition to KIIs, four FGDs (two with rural women only and two with urban women only) were also conducted. The diversified participants of the FGDs were recruited from women of reproductive age groups (15–49 years). Accordingly, pregnant women, women who recently gave birth (< 6 months after giving birth), and women planning to become pregnant were recruited to participate in focused discussions in order to obtain information from different perspectives. The recruitment was held 4 days before data collection. The sites of the meeting (locally known shade of trees) were selected with the study participants considering the distance from their home and convenience for discussion. The date and time of the meeting were also decided by the study participants. Of a total of 44 women approached to take part in the discussions, 38 of them participated. In the first FGD, 10 women (3 pregnant women, 3 women who recently gave birth, and 4 women planning to become pregnant) participated. In the second FGD, 10 women (4 pregnant women, 2 women who recently gave birth, and 4 women planning to become pregnant) participated. In the third FGD, 9 women (2 pregnant women, 3 women who recently gave birth, and 4 women planning to become pregnant) participated. Lastly, in the fourth FGD, 9 women (3 pregnant women, 3 women who recently gave birth, and 3 women planning to become pregnant) participated.

In general, a total of 51 individuals (*n* = 51): 13 from KIIs and 38 individuals from the 4 FGDs participated in this study. The author identified and recruited the key informants and participants of the FGDs by obtaining support from Ganda leaders and health extension workers. The role of Ganda leaders and health extension workers was only providing information for the author on how to get the study participants; otherwise, they did not participate in the selection of the participants.

### Data collection

Data were collected using semi-structured interview guides which were developed first in the English language and then translated into Afan Oromo and back-translated into English by an author. Two interview guides were developed: One for women and the other for health care providers and health extension workers. The guides were co-designed with consumers and stakeholders. It was prepared by making elicitation with five women and two health care providers. In addition, a detailed discussion was made among the authors considering the research question during the preparation of the guides. The guides have open-ended questions (the sequence of questions moved from general to specific) customized as per the respondent type. It was prepared to address: a) background information of participants, b) individual (women and husband) related barriers, c) community-related barriers, d) health care providers related barriers, e) health extension-related barriers and f) media-related barriers.

To maintain consistency, only one interviewer (investigator) conducted all KIIs, and all the FGDs were conducted by the same facilitator and note-taker. On the day of the discussion, the facilitator and note taker arrived early at the FGD site, arrange the sitting, well come and warmly greet the participants, and create rapport. KIIs were conducted for a duration of 37–54 min, whereas FGDs ran within the range of 78–105 min. Both the KIIs and FGDs were conducted until saturation was reached and no new information was obtained. All interviews and focused discussions were conducted in the Afan Oromo language and recorded using a voice recorder. In addition, field notes were also taken throughout all the interviews and discussions.

### Data analysis

The audio data were transcribed verbatim and translated from the Afan Oromo to the English language by the authors. To ensure accuracy, the translated data were reviewed and compared with the transcribed data. Then, the translated data were entered into Atlas ti version 7.0.71 software for data management and analysis. An inductive process of thematic analysis, as described by Braun and Clarke [25], was employed to identify sub-themes and themes within the data. The analysis started while data collection was ongoing; hence, initial findings were explored in more detail in subsequent interviews and focused discussions. It continued in an iterative process until all key themes were saturated and no new information emerged from the interviews and focused discussions. The investigators read and reread all the transcripts and listened to the recorded audios several times to become familiar with the data. Meaning units, which are key phrases in the text, were identified and outlined. The codes were then ascribed to each meaning unit. To increase rigor, each transcript was coded line by line independently by the first coder and verified by all authors. For codes where discrepancies of coding occurred, an agreement of interpretation was reached through discussion. Then, codes were collated into meaningful groups and patterns to identify sub-themes and major themes. Direct quotes were selected to illustrate the interpretations.

### Trustworthiness

The credibility, dependability, transferability, and confirmability of the study were insured by different techniques. The study gathered data from different perspectives by involving different key informants and participants of FGDs. Multiple authors were involved in data collection and analysis. The authors made summarization at the end of each key informant interview and focused group discussions. In addition, the transcribed data were returned to the study participants for commenting and making sure that the data was their own words and ideas. The authors also stayed in the field all during the period of participants recruitment, data collection, and member checking. Indeed, detailed methodologies and research processes were documented and used to assist interpretations of the findings. The analyzed and interpreted data were tested against audio tapped data and field notes before producing the final report. Furthermore, Colleagues were asked to comment on the analyzed and interpreted data. The findings of this study were audited and verified by a research assistant, colleagues, and other experienced individuals in qualitative research.

### Ethical consideration

The study was approved by the Institutional Review Board of Jimma University. The necessary permission was obtained from the district health office and Ganda administrative offices.

All the study participants were informed about the purpose of the study and that they could opt-out of the research at any stage without suffering any negative consequences. Refusal to participate or to continue did not lead to any loss of personal benefit. Written informed consent was obtained from all participants before conducting the interviews and discussions. In addition, permission to audio-record conversations was obtained before data collection. The confidentiality of the participants was kept by using pseudonyms. All personal identifiers were removed from the transcripts and quoted texts.

## Results

### Background information on the study participants

A total of 13 KIIs and 4 FGDs were conducted among different individuals. Thirty-eight women of reproductive age groups participated in the 4 focused group discussions. The mean age of the reproductive age group women was 29.67(range:25–38). The majority of the women were illiterate in terms of their educational status and multiparous in terms of parity. (Table 2).

**Table 2:** Background information of the study participants

Key Informant Interview	Informants			Residency		Age (yrs.)	Education		Total
				Rural	Urban				
	Health care providers			2	4	26–30	Tertiary		6
	Health Extension workers			1	1	24–26	Tertiary		2
	Traditional birth attendant			1	–	35–40	Primary		1
	Pregnant women			2	–	29–30	Primary		2
	Woman who had faced adverse pregnancy outcome			–	1	35–40	Primary		1
	Grandmother			–	1	55–60	Secondary		1
	Total key informants								13
**Focused Group Discussion**	**Participants**	**Residency**		**Age (yrs.)**	**Education**		**Parity**		**Total**
					Illiterate	Primary and above	Primpara	Multipara	
		Rural	Urban						
	Pregnant women	6	6	25–35	5	7	5	7	12
	Women planning to become pregnant	8	7	24–38	7	8	7	8	15
	Women who recently gave birth	6	5	25–32	6	5	5	6	11
	**Total Participants of FGDs**								**38**

### Barriers to the uptake of preconception care

The analysis of the data identified several barriers to the uptake of preconception care, and organized into five major emergent themes: (1) women-related barriers, (2) husband-related barriers, (3) community-related barriers, (4) health service-related barriers, and (5) media-related barriers (Table 3).

**Table 3:** Themes and sub-themes on barriers to the uptake of preconception care

Themes	Sub-themes
Women related barriers	Knowledge
	Belief and fear
	Unplanned pregnancy
	Workload
Husband related barriers	Husband support
Community-related barriers	Perceived benefits
	Culture
Health service-related barriers	Economy, cost/service charge, and distance
	Unavailability of services and shortage of supplements
	Disrespect, humiliation, and abuse
	Knowledge of health care providers
Media related barriers	Attention from media personnel

### Women related barriers

#### Women’s knowledge

Almost all key informants and participants of the FGDs described that women’s lack of knowledge about PC was the main barrier to the uptake of preconception care. They explained that the majority of women turned to health facilities only after confirmed pregnancy or an unsuccessful attempt of conception. They also pointed out that women do not gather information about this issue since they have no awareness.

*“They do not prepare for pregnancy because they do not know” (KII, Health care provider).*

*“.... This is due to a lack of knowledge. Those who have knowledge can prepare for pregnancy” (FGD, >30 years, Woman who recently gave birth).*

The majority of the study participants explained that women use their grandmothers as a reference to do something. They share their grandmothers’ experiences. Some women also give focus on the birth they gave previously and conclude that attending health care facilities before conception is not important because they previously gave normal birth without attending preconception care.

*“Women do not prepare for their becoming pregnant because they say our grandmothers also gave birth without getting this care. Some women may say, I gave the previous births without getting any care before I conceive. I did not face any problems. Therefore, it is not needed” (FGD, 20-30 years, Woman planning to become pregnant).*

*“We usually rely on our grandmothers. We do not think it is important because our mothers/ grandmothers even for conception, gave all births at home without any preparation” (KII, 20-30 years, Pregnant woman).*

#### Belief and fear

The majority of the key informants and participants of FGDs cited that some women belief conception is a natural event and does not need to consult health care providers before it occurs. They believe that a normal fetus is given by God/Allah, not due to the care that women get from health care providers before the occurrence of conception. Participants also explained that women perceive that they are healthy and do not need to go to the health facility without feeling ill, and believe that any care related to pregnancy is needed after the fetal movement started.

*“...Some women say, health is at the hand of the God/Allah and no need of attending the health care facilities before the occurrence of conception” (KII, Health care provider).*

“*They consider as they are healthy unless they feel some illness and do not go to the health facility before they conceive” (KII, Health extension worker).*

*“I do not think attending health facility is needed for the sake of becoming pregnant unless there are some problems. Care and services related to pregnancy are needed after the fetal movement started” (FGD, 20–30 years, Woman who recently gave birth)*

One participant of the FGD explained that fear of positive results was one of the deterrent factors for using preconception care. She felt that some women fear positive results to be screened for different diseases.

*“...The other reason may be due to fear of positive results. For example, women may fear a positive result of HIV testing. Some women may think as no survival after positive result” (FGD, >30 years, Woman planning to become pregnant).*

#### Unplanned pregnancy

The majority of the key informants and participants of the FGDs described that unplanned pregnancy is one of the factors that hinder women from preparing for pregnancy. They repeatedly mentioned that unplanned pregnancy was a common problem in the community. The participants explained that some women do not know as they conceived and they aware of it while they visit health facility for other services.

*“...It is due to an unplanned pregnancy. For some women, this may be due to menstrual disturbance. For example, women who know about preparation for pregnancy but her menstrual period is disturbed may conceive without getting this care” (FGD, >30 years, Woman planning to become pregnant).*

*“The majority of women do not do anything before they conceive. Some of them even do not know as they are pregnant. They aware of it after the fetal movement started. Some of them hear as they get pregnant while they attend health facility for some illnesses. They also give birth without knowing their gestational age” (KII, 20-30 years, Pregnant woman).*

#### Workload

The study participants described that some women were not using PC due to workload. Workload prevents women from giving attention to their health and services utilization. They explained that women in the study area were more responsible for fulfilling their families’ needs like food than their husbands, and this makes the difficulty of preparing for pregnancy, especially for those who had many children.

*“In this area, almost in all households, women are the one who take responsibility for paying for materials and food needed for the family members. They are busy with work and do not contact health care providers for services.” (KII, Traditional birth attendant).*

“*Women, especially those who had many children were usually busy with work. They also do not give attention to their health because they think about their children and give priority to them. For instance, they do not eat a balanced diet, rather they give for their children” (FGD,>30 years, Woman who recently gave birth).*

### Husband support

The study participants described that husband support has a great place in women’s service utilization. However, in the study area, husbands were the major decision-makers in all activities of the families, and the majority of them did not support their wives to attend health facilities. Many of the key informants and participants of the FGDs explained that the majority of husbands in the study area felt superior to their wives and did not have joint discussions with their wives. They highlighted that sometimes the spouses did not reach a consensus even for having or not having a child.

*“...The relationship between the spouse is very important. Despite this, the majority of the husbands do not support their wives, especially in rural areas. Even, they may not give money for their wives to go to health facilities” (FGD, 20-30 years, Pregnant woman).*

*“....It can be due to lack of husband support. This is common in our community. Many couples do not have a commitment. Sometimes husband may need to have a child, but wife may not need or vice versa. I also faced this problem. Most women are under the influence of their husbands. They should discuss and reach at consensus before she conceives and the husband should support his wife....” (KII, Woman who faced adverse pregnancy outcomes).*

### Community-related barriers

Some of the study participants explained that the communities’ perception of care before conception as being less important than other services like antenatal care and skill delivery was one of the identified barriers to the utilization of preconception care. They stated that due to the perception of less importance, people do not set an agenda and share information about this issue.

*“...The other reason is due to lack of sharing information about the importance of preparing for pregnancy among people such as one to five network members” (FGD, 20-30 years, Woman planning to become pregnant).*

*“The community does not think it is important and does not encourage women to prepare for pregnancy” (KII, Health Extension worker).*

Culture also has a great impact on maternal and child health. Some of the key informants described that culturally talking about the desire to become pregnant is considered a shame and should be kept secret. They felt that even talking about becoming pregnant, after conception by itself, women should wear wider clothes to keep secret their pregnancy during the first few months until miscarriage is ruled out.

*“.... Women do not say I want to become pregnant. It is a shame to talk about the desire for getting pregnant and considered as breaking the communities’ norm. Even after conception, women do not want to talk about pregnancy during the first five months which is due to fear of abortion” (FGD, >30 years, Woman who recently gave birth)*

One key informant described that the community of the study area had a negative perception of having many children and narrow birth intervals. She pointed out that this has an influence on the women to prepare for pregnancy and afraid to talk to others about their desire of becoming pregnant.

*“Some of the women do not want to tell and show as they are pregnant because they fear that people are saying she is becoming pregnant again and again but she has a lot of children and even she is unable to grow them. Others may also think as people are saying, she gave birth recently but she is pregnant again” (KII, 20-30 years, Pregnant woman).*

### Health service-related barriers

#### Economy, cost / service charge, and distance

Findings of this study indicated that low households’ income, cost of service, and distance from health facilities were one of the identified barriers to the uptake of preconception care. Many of the key informants and participants of the FGDs suggested that the majority of households are poor and women do not go to health facilities because of the cost of services such as for investigations and medications. The study participants felt that even for wellness checkups, they did not go to the health facility when they get sick.

*“...The main reason is due to lack of economy. Women do not have enough money to pay for checking up their health status. Even when they get ill, they do not go to the health facility unless it is very severe due to lack of money.” (KII, Grandmother).*

*“The health facilities ask money for card, laboratory, and medications. Because of these women do not attend health facilities for the sake of becoming pregnant” (FGD, 20-30 years, Woman who recently gave birth).*

*“...This can be due to lack of money; otherwise, women can prepare before getting pregnant” (FGD, 20-30 years, Woman planning to become pregnant).*

One participant of the FGD described that a long distance from the health facility, coupled with cost of transportation, deters women from getting services before their conception.

*“Distance from the health facility is also one of the reasons for not attending care before getting pregnant. Women travel for three or four hours to reach a health center. There is also a payment for transportation. All these had an influence on visiting health facilities” (FGD, >30 years, Woman planning to become pregnant).*

#### Unavailability of services and shortage of supplements

The majority of the key informants explained that services like screening for some diseases are not available at health facilities nearest to the community. The key informants also repeatedly described that the shortage of supplements like iron and folic acid was one of the main barriers.

*“...the other reason is due to the unavailability of the services. For example, women should go to the hospital for cervical cancer screening because it is not available at health centers. People do not want to be referred to hospital for screening*. *The government should avail the materials and services needed for this care” (KII, Grandmother).*

*“We are giving iron and folic acid only for pregnant women due to the shortage of these supplements. But it is better if given for all women. Sometimes we prescribe to private pharmacies, for those who able to buy” (KII, Health care provider).*

#### Disrespect, humiliation, and abuse

The participants of the study described that disrespect and abuse from health care providers are not only barriers to preconception care but also for other services. They felt that some health care providers reflect bad habits like shouting, scolding, threatening, insulting and ignoring or abandoning clients, which prevent clients from visiting health facilities.

*“Some health care providers insult and shouting at clients. This is also one reason. I myself do not want to go to this health center because they insult us. For instance, I am following my antenatal care visits at another health facility (FGD, >30 years, Pregnant woman).*

*“......Some health care providers do not respect their clients. Women who faced disrespect during some services like antenatal and delivery can never visit the health facility for the sake of getting pregnant. (KII, Health extension worker).*

#### Knowledge of health care providers

The key informants, for instance, health extension workers and some health care providers frequently stated that they did not raise PC with women because of a lack of detailed information about this issue. They felt, even creating awareness for the community, the majority of health care providers by themselves do not know this issue.

*“......health workers also do not know this issue. For example, if a woman goes to health centers, they consider as she comes for illness, not for preconception care” (KII, Health extension worker).*

*“We are not telling about preconception care to the people. I myself have no detailed information about this issue” (KII, Health care provider).*

### Media related barriers

The other identified barrier was the media. The majority of the study participants explained that media personnel gave less attention to the transmission of information about preconception care.

*“Media (Television and radio) are transmitting different programs like family planning and delivery. However, I haven’t been heard anything about PC from media” (KII, Health care provider).*

*“We learned a lot about the negative consequences of home delivery from television. However, it is not transmitting information about preparations before conception” (FGD, > 30 years, Woman planning to become pregnant).*

## Discussion

Our study identified several barriers related to the uptake of PC in the Mana district, Jimma zone, Oromia region, Ethiopia. The biggest barrier we identified was women’s lack of knowledge about preconception care. Other barriers to the uptake of PC were beliefs and fear; unplanned pregnancy; workload; lack of husband support; culture; economy, cost/service charge and distance from health facility; unavailability of services and shortage of supplements; disrespect, humiliation, and abuse; health care providers lack knowledge; and poor media attention to PC.

There are several women’s related barriers to the uptake of preconception care. Among these, women’s lack of knowledge about the benefits of preconception care is a major deterrent to the uptake of PC. Similarly, other studies from high-income countries [26–31], upper-middle-income countries [32, 33], and low-income countries [34] found this as a key barrier to the uptake of preconception care. This was due to the fact that a new program in many countries and the existence of knowledge gaps among health care providers. It was also due to less attention of media on creating communities’ awareness of this issue. Lack of knowledge can deprive health-seeking behavior and service utilization. This suggests the need for developing communication and marketing strategies for increasing awareness for the community. Advertising campaigns, perhaps using novel methods such as social media, should be emphasized.

In the current study, women also perceived that conception is a natural event and no need to attend health facilities before it occurs. They believed that conception and getting a normal fetus is at the hand of Allah /God rather than care given at health facilities. This issue has also been discussed in the literature from the Netherlands [35], Italy [28], and Australia [30]. There is a need for collaboration between health institutions and religious organizations. The findings of the current study also showed that women believed as they are healthy and do not need to attend health facilities for the sake of becoming pregnant unless they get an illness, which is in agreement with studies from the United Kingdom [36], Netherlands [35] and Malaysia [33]. This finding demonstrates how one’s perception of illness affects the decision to seek care. Health education and counseling on the importance and benefits of preconception care for both maternal and child health should be emphasized. The test results also determined women’s uptake of preconception care. The fear of positive test results also prevented women from visiting health facilities for the uptake of preconception care. This was due to stigma and discrimination related to different diseases. A study from Zimbabwe also highlighted this fear of positive results [34]. Encouraging women about the benefits of screening and reassuring the confidentiality of test results could help to break their fear.

The current study as well as earlier studies [26, 29, 33, 36, 37], recognized unplanned pregnancy as an important deterrent to the uptake of preconception care. These findings implicated unplanned pregnancy remains affecting maternal and child health. Women, especially those living in rural areas, are too late (a few months after conception) to aware of their pregnancy. This was due to the lack of using family planning or irregularity of the menstrual cycle as a side effect of family planning. There is a need for strengthening awareness creation on the utilization of family planning by health extension workers during home to home visits, conferences, and outreach activities. Internal linkage, counseling, and client-centered method selection should also be emphasized at all health facilities. This study also found that workload as one of the major barriers to the uptake of preconception care. Women in the study area did not go to a health facility for PC care because they remained engaged in household chores throughout the day to meet their basic daily needs and of children and husbands. Fulfilling families’ need were the responsibility of women than their husbands. Similar findings have been observed in studies conducted in Ireland [31] and Malaysia [33]. The time women spend on performing their daily activities prevents them from traveling to healthcare facilities. Putting responsibility on women for daily basic needs in the other word implies the existence of gender inequalities. This calls for strengthening gender mainstreaming.

Husbands had a great role in women’s and children’s health. Women who were able to decide themselves or jointly decide with their husbands were more likely to use preconception care than those who were not able to decide themselves [24, 38]. However, in the current study, even though women were the major responsible individuals in fulfilling the family’s needs, husbands were the main decision-makers in all activities and did not support women to obtain health services. Similar findings have been observed in studies from developed countries [29, 39] and developing countries [32–34]. This implies the existence of gender inequality all over the world. Gender inequality is common especially in developing countries like Ethiopia where almost all of the households are headed by men and resource allocation is controlled by husbands. A potential solution to this problem is creating awareness about the health consequences of power dominancy. In addition, there is a need to informing and sensitizing women of reproductive age and advocating husband’s support on preconception care [38]. Promotional messages should also be focused on highlighting the impact of not using preconception care.

Culture affects service utilization. It was reported as barriers for up taking preconception care. This study found that culturally talking about the desire for becoming pregnant was considered as breaking norm and it is shameful. Most women kept their pregnancy intent secret between their partner and themselves. Even talking about the desire for becoming pregnant, women did not reveal early pregnancy to the public. This was due to the community perception that talking about the desire of becoming pregnant or pregnancy is a shame until miscarriage is ruled out. This finding is in agreement with a study conducted in the United Kingdom, Netherland, Australia, and Zimbabwe [29, 34–36]. This puts pressure on women to miss preconception care and too late to attend antenatal care which implies how much miscarriage is common and affecting the maternal and child health. It is important to consider how these social norms can be addressed. There is a need for creating awareness through media campaigns using information education communication materials and counseling potential women during health visits for other services.

Failure to utilize PC was further complicated by low household income coupled with the cost of accessing services and distance from health facilities. Low incomes meant many women could not afford the cost of accessing services and transportation. This is challenging especially for rural residents which requires long distances travel to reach health facilities. These findings were supported by different studies [27, 29, 31–34, 36] The implication is that even when women recognize the need to and want to access care, the inability to overcome the service charge and cost of transportation constitutes another challenge. This barrier is solved by strengthening the existing facilities and strategies. The existence of health centers and health posts that are nearest to the community, and health insurance are good opportunities to solve these problems.

Unavailability of services and shortage of supplements were also the identified barriers to uptake preconception care. Others have also reported similar findings in their studies [27, 32, 33]. Unavailability of services implies that all components of PC were not focused. There is a need for the establishment of preconception care strategies that can address all the components of the care at all levels of health care facilities. The shortage of supplements in the current study is due to frequent stock out and the provision of supplements on a quota system not according to actual demand. It will be necessary for health facilities and management teams in the Mana district to improve and strengthen their logistic systems.

The current study, in line with studies from the Netherlands, Italy, and Iran [28, 32, 35] found disrespect, humiliation, and abuse from health care providers as a barrier for the uptake of preconception care. Callous services by health care providers lead customers not to visit health facilities. Health care providers need to be trained on the importance of empathy towards clients and courteous behaviors. This helps to improve the responsiveness of health care providers and benefits users to easily access health services. There is also a need to strengthen management found at all levels. This study also found health care providers’ lack of knowledge about preconception care as barriers to its uptake. This finding was supported by studies conducted in the United Kingdom, Netherland, Ireland, Malaysia, and Iran [26, 31–33, 40]. The current and previous studies showed that some health care providers were not familiar with this issue. There is a need to provide training and mentorship for health care providers especially, for those who work on maternal and child health care to mitigate this barrier. Our study also identified that less attention of media personnel on transmitting information about preconception care deterred women from utilizing preconception care. This issue was also discussed in the study conducted in Italy [28]. There is a need for transmitting information about preconception care using media methods to reach a large segment of a population at a low cost.

### Strengths and limitations

This study has several strengths. This is the first study in Ethiopia that identified barriers to the uptake of preconception care. A variety of key informants and a diverse group of participants in the focused group discussions were included in the study for a holistic understanding of barriers related to the uptake of preconception care. The focused groups allowed frank discussion among attendees. The data were collected until saturation was reached. The findings of this study significantly help for the development of locally appropriate and acceptable programs aiming to improve the utilization of preconception care. However, this study does not end without limitations. Despite its importance, husband views were not included in this study due to a shortage of resources. Hence, further investigation is needed to examine this issue. There might be the probability of the occurrence of bias during the recruitment process of the study participants. In addition, participants’ responses to the study questions might be subject to bias, including social desirability bias. To mitigate this possibility, the study participants were asked to consider not only their perspectives but also the perspectives of others they may have heard about. Indeed, the difficulty of prioritizing the barriers for intervention is another limitation of this study. Furthermore, literatures relating to this specific title in developing countries were very limited.

## Conclusion

This study identified a variety of barriers to uptake of preconception care from both the demand and supply sides which include women, husbands, community, health service, and media related barriers. Knowledge gaps were noted from both the consumers and providers’ sides. These findings imply the importance of scaling up health education and counseling, addressing gender inequalities, and providing customers friendly health services, and establishing preconception care strategies and functional units that can address all the components at all levels of health care facilities. Therefore, health promotion programs are recommended to improve the communities’ knowledge and awareness about PC. This can be achieved by strengthening the health extension programs and through the provision of counseling on PC for all reproductive age groups attending health facilities for any services. We also advise the involvement of community members such as religious leaders and other significant others in designing and implementing health programs. Extensive training on capacity building and necessary attitude and behavioral changes of health professionals are highly recommended to improve the uptake of preconception care**.** To mitigate physical and economic barriers, linking PC to the existing strategies like community-based health insurance scheme or providing free services for all women of reproductive age groups are advised. We also recommend availing PC services at all possible health facilities nearest to the communities to solve physical constraints and the cost of transportations. In addition, researchers also advised to conduct studies on issues related to preconception care, especially in developing countries, including Ethiopia.

## Supplementary information

**Additional file 1.** Interview Guide for Health Care Providers and Health Extension Workers

## Data Availability

The data of the study are available from the corresponding author upon reasonable request.

## Abbreviations

AIDS: Acquired immune deficiency syndrome
FGD: Focused group discussion
HIV: Human immune virus
KII: Key informant interview
PC: Preconception care
STI: Sexually transmitted infections

## Footnotes

1 **Ganda:** the lowest government administrative hierarchy that exists next to district
