# Supplementary material for: Why do women not prepare for pregnancy? Exploring women’s and health care providers’ views on barriers to uptake of preconception care in Mana District, Southwest Ethiopia: a qualitative study
Source: BMC Pregnancy Childbirth. 2020 Sep 1;20:504. doi: 10.1186/s12884-020-03208-z (PMC7465426; doi:10.1186/s12884-020-03208-z)
Supplement: Supplementary file 1 — Additional file 1. Interview Guide for Health Care Providers and Health Extension Workers [file 12884_2020_3208_MOESM1_ESM.docx]

**Interview Guide for Health Care Providers and Health Extension Workers**

| **Background information** | |
| --- | --- |
| Age |  |
| Sex |  |
| Residency |  |
| Educational level |  |

**Guiding questions:**

1. What are the services that your health facility is providing for non-pregnant women?

2. Was there anything that you advise women to do/not to do before they become pregnant? **Probe:** on what issues? What do you advise them?

3. Did you ever ask the women who came for other services whether they are considering pregnancy in the near future or not? **Probe**: Who do you ask? When do you ask? For what purpose do you ask?

4. What do the women planning to become pregnant should do before they conceive? **Probe**: It can be anything (preparation) they do at their home; can be services they get from the health institutions, communities, or others?

5. How do you understand preconception care? **Probe**: What are the services provided during preconception care? How should provide the services? Where it should be provided? For Whom?

6. What do the women planning to become pregnant are practically doing in your community before they get pregnant? **Probe**: It can be anything (preparation) they do at their home; can be services they get from the health institutions or others?

7. How do the communities in your area think/believe about care before pregnancy? **Probe:** Anything they do before they get pregnant? For what purpose they do?

8. Why women are turning to the health facility only after they get pregnant? i.e. why they do not prepare for pregnancy before they conceive? **Probe**: Can be women related issues, husband related issues, friends/family-related issues, communities related issues, health care providers related issues, health extension worker-related issues, media-related issues, etc? Can you explain with examples without mentioning their names?

9. Who do you think have the role to improve preconception care? **Probe**: Explain their roles?

**Interview Guide for Women**

| Participants’ Code | Age | Residency | Educational level | Parity |
| --- | --- | --- | --- | --- |
| P1 |  |  |  |  |
| P2 |  |  |  |  |
| P3 |  |  |  |  |
| P4 |  |  |  |  |
| P5 |  |  |  |  |
| P6 |  |  |  |  |
| P7 |  |  |  |  |
| P8 |  |  |  |  |
| P9 |  |  |  |  |
| P10 |  |  |  |  |
| P11 |  |  |  |  |
| P12 |  |  |  |  |

**Guiding questions:**

1. What services given for the non- pregnant women do you know?

2. What do the women planning to become pregnant should do before they conceive? **Probe**: It can be anything (preparation) they do at their home; can be services they get from the health institutions, communities, or others?

3. What do the women planning to become pregnant are practically doing in your community before they get pregnant? **Probe**: It can be anything (preparation) they do at their home; can be services they get from the health institutions or others?

4. From your experience, what things you were doing when you want to get pregnant? **Probe**: What makes you to do so? Who supported you?

5. What do you think the benefit of care before pregnancy: **Probe**: For women? For family? For community? For county? How?

6. How do the communities in your area think/believe about care before pregnancy? **Probe:** Anything they do before they get pregnant? For what purpose they do?

7. Why women are turning to the health facility only after they get pregnant? i.e. why they do not prepare for pregnancy before they conceive? **Probe**: Can be women related issues, husband related issues, friends/family-related issues, communities related issues, health care providers related issues, health extension worker-related issues, media-related issues, etc? Can you explain with examples without mentioning their names?

8. Who do you think have the role to improve preconception care? **Probe**: Explain their roles?

**Thank you for your time and ideas!!!**
